# Bifunctional MOF‐on‐MOF‐Derived CuCo_2_O_4_ for Oxygen Evolution Reaction Electrocatalysis and Supercapacitor Electrodes

**DOI:** 10.1002/open.202500180

**Published:** 2025-07-31

**Authors:** Johnnys da Silva Hortêncio, Rafael A. Raimundo, Allan J. M. Araújo, André Luiz Menezes de Oliveira, Daniel A. Macedo, Sherlan Guimarães Lemos, Fausthon Fred da Silva

**Affiliations:** ^1^ Departamento de Química Universidade Federal da Paraíba (UFPB) João Pessoa PB 58.051‐900 Brazil; ^2^ TEMA ‐ Centre for Mechanical Technology and Automation Department of Mechanical Engineering University of Aveiro 3810‐193 Aveiro Portugal; ^3^ LASI ‐ Intelligent Systems Associate Laboratory 4800‐058 Guimarães Portugal; ^4^ Núcleo de Pesquisa e Extensão LACOM Universidade Federal da Paraíba Joãao Pessoa PB 58051‐900 Brazil; ^5^ Laboratório de Síntese Química de Materiais Dept. de Engenharia de Materiais Universidade Federal do Rio Grande do Norte Natal RN 59078‐970 Brazil; ^6^ Programa de Pós‐Graduação em Ciência e Engenharia de Materiais ‐ PPCEM Universidade Federal da Paraíba (UFPB) João Pessoa PB 58.051‐900 Brazil

**Keywords:** copper cobaltites, metal–organic frameworks, oxygen evolution reaction, supercapacitors, water splitting

## Abstract

The development of high‐performance electrocatalysts for oxygen evolution reaction (OER) is still a challenge to produce green hydrogen. Thus, herein, a new bifunctional metal–organic frameworks (MOF)‐derived CuCo_2_O_4_ is obtained, applied as OER electrocatalyst and electrode for supercapacitors. All physicochemical and morphological characterization indicates the formation of a pure spinel structure CuCo_2_O_4_ crystalline phase and coral reef‐like morphology. X‐ray photoelectron spectroscopy data showed major presence of Co^3+^ and Cu^+^ ions on the surface and high concentration of oxygen vacancies. OER electrocatalytic assays conducted in alkaline medium (1.0 M KOH) show a reduced overpotential (*η*) of 317 mV at 10 mA cm^−2^ and Tafel slope of only 49 mV dec^−1^, besides excellent electrochemical stability up to 12 h. The material is also studied for supercapacitors applications via cyclic voltammetry (CV) and galvanostatic charge–discharge (GCD) analysis. CuCo_2_O_4_ material presents specific capacity near 75 C g^−1^, at least ≈2.8 times higher than pristine CuO and Co_3_O_4_ at 1 A g^−1^. This results indicate the MOFs‐derived CuCo_2_O_4_ as a promising bifunctional material for energy conversion and storage.

## Graphical abstract

A bifunctional copper cobalt oxide derived from metal‐organic frameworks was synthesized with a spinel structure and coral reef‐like morphology. It showed excellent activity for the oxygen evolution reaction, with overpotential of 317 mV at 10 mA cm^−2^ and Tafel slope of 49 mV dec^−1^. As supercapacitor electrode, it delivered 75 C g^−1^, outperforming copper and cobalt oxides significantly.

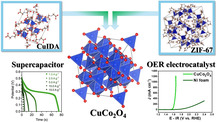

## 1 Introduction

The increasing demand for renewable and sustainable energy storage and conversion technologies has accelerated the development of new methods to reduce fossil fuel consumption and environmental pollution. In this context, supercapacitors and electrocatalytic water splitting are focused on recent literature, as good candidates to replace fossil fuels.^[^ ^1^ ^,^ ^2^ ^]^ Supercapacitors (SCs) stand out due to their excellent stability, high power density, and fast charge–discharge characteristics. They are applied in hybrid vehicles, smart grids, industrial‐scale power generation, portable electronics, and various power management devices.^[^ ^3^ ^,^ ^4^ ^]^ On the other hand, new earth‐abundant based electrocatalysts are the key‐issue to solve the energetic problems of hydrogen production.^[^ ^5^ ^]^ Water electrolysis occurs through two electrodic reactions named hydrogen evolution reaction (HER, cathodic process) and the oxygen evolution reaction (OER, anodic process). The OER (alkaline medium: $4{\mathrm{OH}}^{-}(\mathrm{aq})⇌2H_{2}O(l)+O_{2}(g)+4e^{-}$) is limited by slow kinetics and high overpotentials; thus, high‐efficient electrocatalysts are requested to improve the reaction rate and reduce the overpotential.^[^ ^6^ ^,^ ^7^ ^]^

Noble metal oxides like RuO_2_ and IrO_2_ have the highest performances as electrocatalysts for OER,^[^ ^8^ ^]^ and Ru‐based materials are considered ideal electroactive materials in SCs due to their high specific capacitance, high conductivity, and good stability.^[^ ^9^ ^]^ However, these chemical elements have some shortcomings such as low abundance and high cost, and materials based on Earth‐abundant like Co, Ni, and Cu hydroxides have attracted attention due to their remarkable electrochemical properties and multifunctional applicability.^[^ ^10^ ^]^ Particularly, spinels such as MCo_2_O_4_ (M = Zn, Ni, Mn, Cu, Fe, etc.) have stood out due to their physicochemical properties and various applications, including supercapacitors (SCs) and the electrocatalysts for OER.^[^ ^6^ ^,^ ^11^ ^]^

Metal–organic frameworks (MOFs)‐derived nanomaterials are promising systems for energy conversion and storage.^[^ ^12^ ^,^ ^13^ ^]^ Notably, Zeolitic Imidazolate Frameworks‐67 (ZIF‐67, cobalt 2‐methylimidazole) stands out as the most explored MOF in the literature to obtain new spinel‐based cobalt containing nanomaterials for energy conversion and storage.^[^ ^14^, ^15^ ^–^ ^16^ ^]^ Recently, our research group has demonstrated the potential of MOFs as precursors for the production of new electrocatalysts in OER, using MOFs such as ZIF‐67 and CuIDA ([Cu (IDA) (H_2_O)_2_], IDA = iminodiacetate).^[^ ^17^, ^18^, ^19^, ^20^, ^21^ ^–^ ^22^ ^]^

On the other hand, copper cobaltite (CuCo_2_O_4_) exhibits excellent conductivity, electrochemical activity, and high specific capacitance when compared to other transition metal oxides such as MnO_2_, V_2_O_5_, and Co_3_O_4_.^[^ ^23^ ^]^ The d‐orbitals of these transition metals are partially filled, which enhances their electrocatalytic activity due to the effective electronic interactions within the material.^[^ ^24^ ^]^ Several studies have been published on the multifunctional application of CuCo_2_O_4_, including supercapacitors, lithium‐ion batteries, glucose sensors, and electrocatalysts for water‐splitting.^[^ ^25^, ^26^, ^27^, ^28^, ^29^, ^30^, ^31^ ^–^ ^32^ ^]^ For example, Pawar et al. synthesized nanoporous CuCo_2_O_4_ nanosheets for SC and OER applications.^[^ ^33^ ^]^ The material had a specific capacitance of 1473 F g^−1^ at 1 A g^−1^ with ≈93% capacitance retention after 5000 cycles. OER analysis revealed an overpotential of 260 mV at 20 mA cm^−2^ with a Tafel slope of 64 mV dec^−1^.^[^ ^33^ ^]^ Wei et al. also prepared Co (II)‐dominant and oxygen‐deficient CuCo_2_O_4_@carbon quantum dots using the hydrothermal method for SC and OER applications.^[^ ^34^ ^]^ Regarding MOF‐derived materials, Ma,^[^ ^35^ ^]^ Saleki,^[^ ^36^ ^]^ and Guo^[^ ^37^ ^]^ reported pure crystalline phase CuCo_2_O_4_ applied in lithium‐ion batteries, hybrid supercapacitors and lithium storage, respectively. However, until this date, there isn’t reports of MOF‐derived CuCo_2_O_4_ as OER electrocatalysts.

Thus, in this work, a new bifunctional copper cobaltite (CuCo_2_O_4_) was obtained from a ZIF‐67/CuIDA composite, applied for OER electrocatalysis and SCs. The material was characterized by X‐ray diffraction (XRD), vibrational spectroscopy (FT‐IR and Raman), scanning electron microscopy (SEM‐EDS), and UV‐VIS absorption spectroscopy. Electrochemical characterization for OER electrocatalysis (in alkaline medium) and supercapacitors were investigated using linear sweep voltammetry (LSV), cyclic voltammetry (CV), electrochemical impedance spectroscopy (EIS), and chronopotentiometry (CP).

## 2 Experimental Section

### 2.1 Materials

Iminodiacetic acid (HN (CH_2_CO_2_H)_2_, 99%), 2‐methylimidazole (C_4_H_6_N_2_, 99%), and methanol (CH_3_OH, 99%) were acquired from Sigma–Aldrich. Copper (II) acetate monohydrate (Cu_2_ (AcO)_4_·H_2_O, 98%) and potassium hydroxide (KOH, 99%) were purchased from Vetec. Cobalt (II) nitrate hexahydrate (Co (NO_3_)_2_·6H_2_O, 99%) was purchased from Dinâmica Química. All chemicals were used without previous purification. Nickel foam (Ni 99.8%, porosity >95%) was purchased from QiJing Ltd., China.

### 2.2 Synthesis of the CuIDA/ZIF‐67 Composite (CuIDA = [Cu (IDA) (H_2_O)_2_]) and CuCo_2_O_4_ Sample

CuIDA MOF was prepared by the reaction between iminodiacetic acid and copper acetate, using crystallization at room temperature, as reported in our previous work.^[^ ^17^ ^,^ ^19^ ^]^ CuIDA/ZIF‐67 composite was synthesized following the literature, using a CuIDA mass equal to 50% of the ZIF‐67 mass, considering the reaction yield of the pure ZIF‐67. The mass ratio was chosen based on previous literature results for the preparation of nanocomposites based on ZIF‐67.^[^ ^18^ ^]^ A suspension was prepared using 68.3 mg of CuIDA, 1.23 mmol (359.0 mg) of cobalt nitrate, and 37.5 mL of methanol. To this suspension, a methanolic solution (37.5 mL of 2‐methylimidazole ligand (9.87 mmol, 811.0 mg) was added, and the system was kept under magnetic at room temperature for 24 h. After this period, the resulting solid was centrifuged at 6000 rpm for 15 min, and the collected crystals were air‐dried.

To obtain the copper cobaltite, the CuIDA/ZIF‐67 sample was placed in a porcelain crucible and then calcined a preheated muffle furnace at 350 °C for 2 h in air, then naturally cooled to room temperature.

### 2.3 Structural, Chemical, and Morphological Characterization

Experimental powder patterns were acquired in a Shimadzu XRD‐6000 X‐ray diffractometer (K*α* (Cu) = 1.5481 Å). Phase identification, crystallite size, and lattice parameters were determined by Rietveld refinement using TOPAS software. Thermogravimetric analysis (TGA) was performed on a Shimadzu thermal analyzer model DTG‐60H from room temperature to 900 °C (rate 10 °C min^−1^) under synthetic air flow (50 mL min^−1^). Infrared vibrational spectra (in KBR pellets) were measured in a Shimadzu IRPrestine21 spectrophotometer between 400 and 4000 cm^−1^. Raman spectra were recorded at room temperature using the LabRAM‐HR Evolution‐HORIBA spectrometer (532 nm laser, from 100 to 3000 cm^−1^). UV‐VIS absorption spectroscopy was performed in a spectral range of 220–1400 nm using BaSO_4_ as the reflectance standard, in a Shimadzu UV‐3600 spectrophotometer. The morphology of the CuIDA/ZIF‐67 and CuCo_2_O_4_ materials was analyzed by scanning electron microscopy (SEM) with a field emission source (FEG, model MIRA3 LMH, TESCAM) coupled to an EDS module (Oxford Instruments, Ultim Max). X‐ray photoelectron spectra were acquired in a X‐ray photoelectron spectroscopy (XPS) spectrometer (ScientaOmicron ESCA+) with monochromatic Al K_*α*_ radiation (*hν* = 1486.6 eV). High‐resolution XPS spectra were obtained at a constant pass energy of 20 eV with 0.05 eV, and the data processing was performed using the CasaXPS software.

### 2.4 OER Electrocatalytic Investigation

Electrochemical measurements were conducted in alkaline aqueous solution (KOH, 1 M, pH 13.6). Working electrodes were prepared in a previous cleaned Ni foam with geometric area of 1 cm^2^, and experimental details can be found in previous works.^[^ ^17^ ^,^ ^20^ ^]^ Besides CuCo_2_O_4_ sample, pristine CuO and Co_3_O_4_ electrodes were also prepared using the same procedure. All electrochemical measurements were performed on an Autolab potentiostat/galvanostat (PGSTAT302N) with a FRA32M module (Metrohm Autolab) in a standard three‐electrode cell at room temperature. A platinum wire and an Ag/AgCl electrode (saturated 3 M KCl solution) were used as counter and reference electrodes, respectively. LSV was performed at a scan rate of 5 mV s^−1^ in the potential range of 0–1.5 V (versus RHE) for OER. All measured potential were converted to Reversible Hydrogen Electrode (RHE) scale applying the equation Nernst equation: *E*_RHE_ = *E*_Ag/AgCl_ + 0.059 ×pH + 0.1976. Electrodic reaction kinetics were evaluated by Tafel equation^[^ ^38^ ^]^: *η* = *a* + *b* log *j*_0_, where *η* is the overpotential, *j*_0_ is the current density, *b* is the Tafel slope, and *a* is a constant. The overpotential was calculated by the equation: *η* = *E*_RHE_ − 1.23 V.

The electrochemically active surface area (ECSA) was obtained by the double layer capacitance (*C*_dl_ = *i*/*v*, where *i* is the charge current and *v* is the scan rate) using CV measurements in the potential range of 0.20–0.25 V with a scan rate ranging from 10 to 100 mV s^−1^. EIS measurements were carried out in the frequency range of 10^−2^ to 10^5^ Hz with a voltage amplitude of 5 mV at three potentials (0.3, 0.5, and 0.6 V). The experimental impedance data were fitted to an appropriate equivalent circuit and a nonlinear least square fitting procedure. Corrections for ohmic resistance were made using the following equation: *E*_corrected_ = *E −* iR, where *E*_corrected_ was the corrected potential (iR), *E* was the potential applied in the experiment, and *R* was the series resistance obtained in the EIS. Chronopotentiometry was also performed to assess the stability of the electrocatalysts in an alkaline medium (1.0 M KOH), current density of 10 mA cm^−2^ for 12 h at room temperature. All measurements were performed in duplicate to ensure the reproducibility of the data.

### 2.5 Electrochemical Characterization for Supercapacitor Applications

For SC applications, the working electrode (CuCo_2_O_4_) was prepared by dispersing 80 wt% of the active material, 10 wt% of carbon black, and 10 wt% of polytetrafluoroethylene (PTFE) (60 wt% in H_2_O dispersion) in isopropyl alcohol and sonicated for 30 min. Subsequently, the resulting mixture was applied to a previously cleaned nickel foam (1 cm^2^). Finally, the working electrode was heated at 70 °C for 12 h in a vacuum oven to remove residual solvent. The mass loading of the electrode material on the Ni foam was about 5 mg cm^−2^. CV and galvanostatic charge–discharge (GCD) tests were carried out to evaluate the electrochemical performance of CuCo_2_O_4_. CV analysis was performed over a potential window of 0–0.6 V at scan rates of 5–100 mV s^−1^. Furthermore, charge–discharge cycling was carried out within a potential window of 0–0.5 V at a specific current of 1.0–15.0 A g^−1^. The specific capacity was calculated according to the galvanostatic charge–discharge curve (GCD) by the following Equation (1) ^[^ ^39^ ^]^

(1) $$Q_{s}=\frac{I\Delta t}{m}$$

where *Q*_s_ (C g^−1^) is specific capacity, *I* (A) is the discharge current, Δ*t* is the discharge time, and m (g) is the mass of the active material. GCD tests were also performed on CuO and Co_3_O_4_ electrodes as reference samples.

## 3 Results and Discussion

### 3.1 CuIDA/ZIF‐67 Precursor

CuIDA and CuIDA/ZIF‐67 composite precursors were obtained according to previous works in literature, using synthesis at room temperature.^[^ ^17^ ^,^ ^18^ ^]^ Experimental XRD pattern of the CuIDA sample (Figure S1, Supporting Information) confirms the formation of [Cu (IDA) (H_2_O)_2_] (CIF 105,855),^[^ ^40^ ^]^ with high correlation between experimental data and calculated pattern from the CIF file. The FT‐IR spectrum of CuIDA confirms the presence of the main signals related to the iminodiacetate ligand (C—H, C=O, and C—N stretching) and coordinated water molecules (O—H stretching).^[^ ^17^ ^]^

The CuIDA/ZIF‐67 composite was obtained by crystallization of ZIF‐67 in the presence of CuIDA MOF, in a mass ratio of 50%, considering the reaction yield of free ZIF‐67.^[^ ^18^ ^]^ The composite´s diffraction pattern (Figure S3, Supporting Information) fits well with the theoretical diffraction pattern for ZIF‐67 (CIF 1,429,244), with no additional peaks.^[^ ^41^ ^]^ The signals related to CuIDA are observed with very low intensity, due to the greater crystallinity and amount of ZIF‐67 in the sample; however, results indicate that CuIDA did not affect the ZIF‐67 crystallization. The FT‐IR spectra (Figure S4, Supporting Information) showed the ZIF‐67 bands, with signals at 3132, 2958, and 2923 cm^−1^ related to C—H asymmetric stretching and CH_2_ asymmetric/symmetric stretching, respectively. The bands located at 1452, 1413,^,^ and 1141 cm^−1^ correspond to C—N bonds in the aromatic ring, while the signal at 426 cm^−1^ indicates the presence of Co—N bonds. The band for the carbonyl group of the imidazolate ligand appears at 1580 cm^−1^. Additional signals at 3429, 1570, and 1385 cm^−1^ correspond to the O—H, C=O, and C—O organic groups of the iminodiacetate ligand in CuIDA. All signals are by literature,^[^ ^17^ ^,^ ^41^ ^]^ confirming the formation of the CuIDA/ZIF‐67 composite. TGA curve (Figure S5, Supporting Information) in oxidizing atmosphere of the composite shows thermal stability up to 350 °C, where complete collapse of the material occurs, resulting in the formation of metal oxide after this temperature.

SEM images (Figure S6a,b, Supporting Information) showed a detailed morphology of the CuIDA/ZIF‐67 composite. Morphological analysis reveals that the dodecahedral shape of ZIF‐67 is preserved even when incorporated with CuIDA, as reported in the literature.^[^ ^42^, ^43^, ^44^ ^–^ ^45^ ^]^ EDS analysis (Figure S6c, Supporting Information) indicated the presence of the main elements (Cu, Co, O, N and C) in the sample. Additionally, the elemental mapping images (Figure S6d–g, Supporting Information) corroborate the homogeneous distribution of Co and Cu, on the CuIDA/ZIF‐67 composite surface.

### 3.2 CuCo_2_O_4_ Material

**Figure 1:**
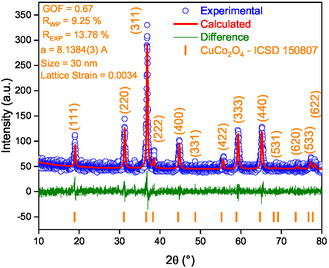
XRD and Rietveld refinement of the synthesized CuCo_2_O_4_.

The calcination of the CuIDA/ZIF‐67 composite resulted in the formation of a single phase CuCo_2_O_4_, as confirmed by powder XRD (**Figure** **1**). The main diffraction peaks observed at 2*θ* = 18.9°, 31.2°, 36.7°, 38.5°, 44.7°, 55.6°, 59.2°, 65.2°, 68 .6°, 74.1°, 77.2°, and 78.2°, correspond to the (111), (220), (311), (222), (400), (331), (422), (333), (440), (531), (620), (533), and (622) planes, respectively. These signals were indexed according to the ICSD 150,807 reference, confirming the crystallization of CuCo_2_O_4_ with spinel cubic structure and Fd‐3m space group. Pure phase of CuCo_2_O_4_ was also obtained by Maji^[^ ^46^ ^]^ and An,^[^ ^47^ ^]^ from Cu‐modified ZIF‐67. Saleki and co‐workers also reported the synthesis of pure CuCo_2_O_4_ from a MOF/MOF ZIF‐67‐based composite.^[^ ^36^ ^]^ Rietveld refinement (Figure 1) indicates the nanostructured nature of copper cobaltite with lattice parameters *a* = *b* = *c* = 8.1384 Å, average crystallite size of 30 nm and lattice strain of 0.0034, and agreement indices (*R*_wp_, *R*_exp,_ and *χ* ^2^) of 9.25%, 13.76%, and 0.67%, respectively. These values indicate a good agreement between experimental data and the fitted model. No substantial changes were observed in the CuCo_2_O_4_ lattice parameters, agreeing with the ICSD card file mentioned above.

**Figure 2:**
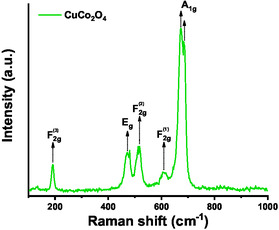
Raman spectrum of CuCo_2_O_4_ spinel nanoparticles.

The MOF/MOF‐derived CuCo_2_O_4_ sample were also characterized by vibrational spectroscopy (FT‐IR and Raman) and results are shown in **Figure** **2** and S7, Supporting Information. FT‐IR spectrum (Figure S7, Supporting Information) showed signals at 568 and 659 cm^−1^, typically associated with the M‐O vibrational modes of the spinel structure.^[^ ^48^ ^]^ The fist signal is related to the Co^3+^–O^2−^ stretching in tetrahedral (*T*_d_) sites, while the late one is due to the Cu^2+^–O^2−^ in octahedral (*O*_h_) sites.^[^ ^23^ ^,^ ^49^ ^]^ Additionally, the band near 3500 cm^−1^ is associated with OH stretching vibrations, indicating the presence of hydroxyl groups on the surface of the CuCo_2_O_4_.^[^ ^50^ ^]^ On the other hand, five active peaks located at 190, 479, 516, 608, and 673/681 cm^−1^ were observed in the Raman spectrum (Figure 2). These signals correspond to the *A*_1g_ + *E*_g_ + 3*F*_2g_ vibration modes of the spinel structure.^[^ ^48^ ^]^

The peak shift observed compared to the free cobaltite is due to the phonon confinement effect in copper cobaltite nanoparticles, leading to uncertainties in the phonon wave vectors and a reduction in the intensity of the Raman peaks.^[^ ^51^ ^,^ ^52^ ^]^ All signals confirm the presence of the spinel structure of copper cobaltite (CuCo_2_O_4_) and agree with similar studies already reported.^[^ ^53^, ^54^ ^–^ ^55^ ^]^ The peak at 190 cm^−1^ is associated with the Co—O stretching mode, related to the tetrahedral Co^2+^‐O^2−^ sites, while the peak at 673/681 cm^−1^ is related to the stretching vibrations of the Cu/Co—O bond in the octahedral sites.^[^ ^20^ ^]^

Pure Co_3_O_4_ obtained under similar conditions from ZIF‐67 showed signals at 191 and 673 cm^−1^, related to the *F*_2g_ and *A*_1g_ vibrational transitions, respectively.^[^ ^20^ ^]^ In this case, the split in the *O*_h_ sites signal obviously indicated the presence of both Co^3+^ and Cu^2+^ ions at this site. However, no changes in the signal for the tetrahedral site were observed, indicating the preference of Cu^2+^ ions for the *O*_h_ sites. These results are similar to UmaSudharshini,^[^ ^56^ ^]^ Mu,^[^ ^57^ ^]^ and Behera,^[^ ^58^ ^]^ for Cu‐doped Co_3_O_4_, several under different experimental conditions. These Raman shifts are observed only at high Cu concentrations in the structure, due to the confinement effect of photons induced by surface oxygen vacancies,^[^ ^57^ ^]^ as confirmed by the XPS measurements described below. The Raman data obtained here are also consistent with the formation of an inverse spinel structure, as described by Angelov and coworkers,^[^ ^59^ ^]^ in which Co^3+^ ions fully occupy the *T*_d_ sites and half of the *O*_h_ sites, while Cu^2+^ ions occupy the remaining octahedral sites, exhibiting local Jahn–Teller distortion.^[^ ^60^ ^]^

The optical properties of CuCo_2_O_4_, prepared at 350 °C, were measured using UV‐VIS absorption spectroscopy (Figure S8, Supporting Information). Three absorption bands between 200–450, 450–1000, and 1000–1400 nm were observed. The first band, in the UV region, is attributed to the ligand‐to‐metal charge transfer process (O^−2^ → M^+x^), while the late ones are related to the d–d electronic transition of the cobalt and copper ions in *O*_h_ or *T*_d_ environment. The optical gap of CuCo_2_O_4_ was calculated using the Tauc relationship (Equation (2)) provided below^[^ ^61^ ^,^ ^62^ ^]^

(2) $$\left(\alpha hv\right)=C{\left(hv-E_{g}\right)}^{n}$$

where *α* is the absorption coefficient, *hv* represents the energy of the incident photon (eV), *C* is a constant, and *E*_g_ corresponds to the energy gap of the material. The value of *n* can be 1/2 for direct transition or 2 for indirect transition.^[^ ^63^ ^,^ ^64^ ^]^ The bandgap can be estimated by extrapolating the linear region of the plot of (*αhv*)^2^ versus *hv* plot, as depicted in Figure S7, Supporting Information. Similarly, the indirect energy gap was obtained through a fitting procedure using the linear portion of the plot (*αhv*)^1/2^ versus *hv* plot, as depicted in Figure S9, Supporting Information. Considering the copper cobaltite as an indirect bandgap semiconductor, the estimated *E*_g_ was 1.14 eV, which is consistent with data found in the literature for materials with similar compositions.^[^ ^65^ ^,^ ^66^ ^]^ This value is lower compared to pure CuO (1.23 eV) and Co_3_O_4_ (1.70 eV), obtained from the CuIDA and ZIF‐67 respectively. This bandgap energy was attributed to the octahedral O^2−^ ions of Co^3+^ and Cu^2+^, as well as charge transfer from O^2−^ to the Co^2+^ energy level in the CuCo_2_O_4_ crystal structure.^[^ ^24^ ^,^ ^67^ ^]^ Compared to pure cobalt oxide, the values obtained for CuCo_2_O_4_ indicate a reduction in the bandgap. As noted by Jibril et al.,^[^ ^68^ ^]^ this smaller gap can contribute to greater oxygen ion mobility within the structure, thereby enhancing the reducibility of the electrocatalyst. Consequently, CuCo_2_O_4_ is expected to exhibit higher electrocatalytic activity.^[^ ^62^ ^,^ ^68^ ^]^

**Figure 3:**
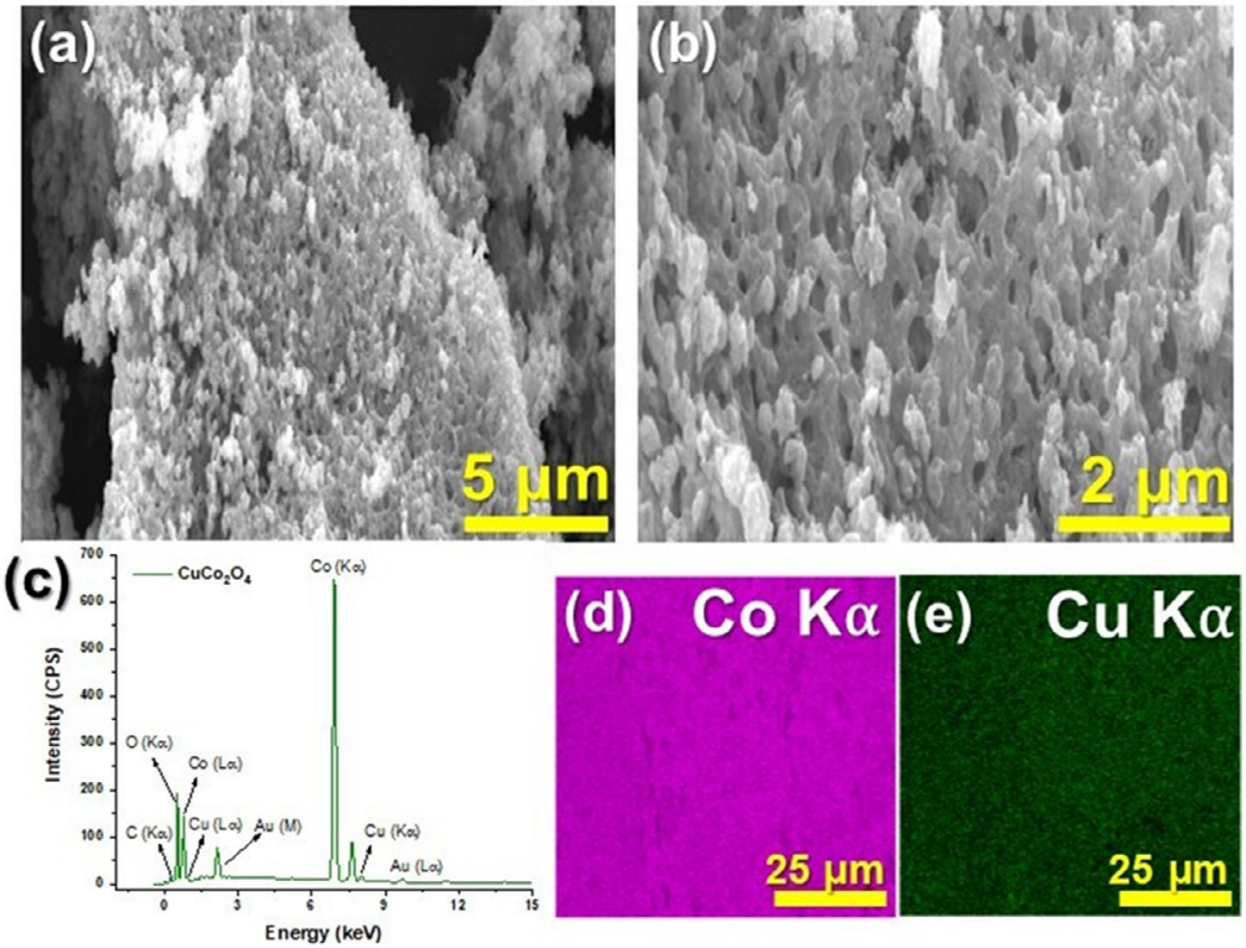
a,b) SEM image of CuCo_2_O_4_ at different magnifications; c) EDS spectrum of CuCo_2_O_4_; d,e) Co and Cu elemental mapping.

The morphology and chemical composition of the CuCo_2_O_4_ electrocatalyst was investigated by SEM‐EDS. SEM images (**Figure** **3**a,b) reveal an irregular morphology, attributed to the calcination process, inducing a quick decomposition of the precursor and subsequent crystallization to form the metal oxide. The nanoparticles tend to aggregate, forming a reef‐like structure, which can provide a large surface area with more accessible active sites and efficient contacts for various reactions.^[^ ^69^ ^]^ EDS analysis (Figure 3c) reveals the presence of O, Co, and Cu.^[^ ^70^ ^]^ Furthermore, the homogeneous distribution of Co and Cu throughout the sample surface is evident in Figure 3d,e.

**Figure 4:**
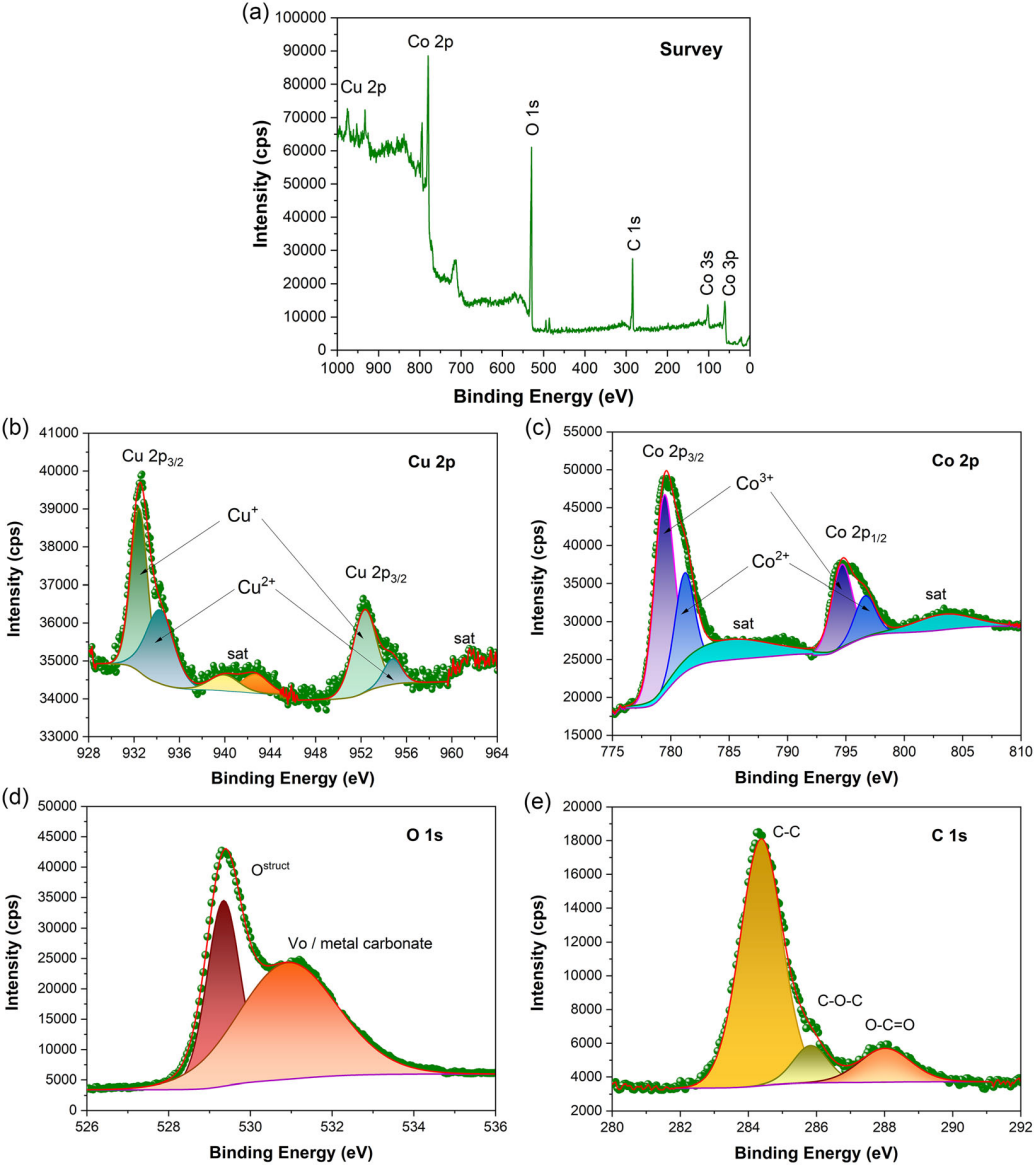
a) XPS survey spectrum, and high‐resolution XPS scans of the b) Cu 2*p*, c) Co 2*p*, d) O 1*s*, and e) C1*s* for the CuCo_2_O_4_ sample.

To better understand the surface characteristics of the CuCo_2_O_4_ sample, XPS measurements were carried out, as shown in **Figure** **4**. The survey XPS spectrum is shown in Figure 4a, and the high‐resolution (HR) XPS scans of Cu 2*p*, Co 2*p*, O 1*s*, and C 1*s* are depicted in Figure 4b–e. Semiquantitative analysis of the sample's surface was performed as listed in **Table** **1**. XPS survey spectrum of the sample revealed that the surface is essentially composed of Cu, Co, O, and C elements (Figure 4a); however, it was noted that a lower Cu concentration is present on the surface compared to Co (Table 1).

**Table 1:** Semiquantitative analysis of the surface composition of CuCo_2_O_4_ sample.

	Cu	Co	O	C
at%	2.30	39.11	28.28	30.31
Std dev	0.25	1.07	0.74	0.88

The high‐resolution (HR) Cu 2*p* XPS spectrum (Figure 4b) was deconvoluted into multiplet splitting related to the Cu 2*p*_3/2_ and 2*p*_1/2_ components, which revealed mixed valences of Cu cations (Cu^+^ and Cu^2+^) and the presence of discreet satellite peaks of Cu^2+^ species. The presence of reduced Cu^+^ species is normally observed in CuCo_2_O_4_,^[^ ^71^ ^,^ ^72^ ^]^ despite some authors deconvoluting the XPS Cu 2*p* spectrum into components only related to Cu^2+^ species.^[^ ^25^ ^,^ ^73^ ^,^ ^74^ ^]^ The difference in the Cu^+^ content observed in the present work compared to the literature is likely attributed to the synthesis method used to obtain CuCo_2_O_4_ material. The deconvolution of the peaks revealed the presence of 30.75% Cu^2+^ ions and 69.25% Cu^+^ ions (**Table** **2**). The predominance of copper ions in the lower oxidation state is consistent with a high concentration of Co^3+^ ions on the surface, due to electron migration toward the copper ions, given the higher electronegativity of this element.

**Table 2:** Deconvolution data of the HR Cu 2p and Co 2p XPS spectra.

CuCo_2_O_4_					
Cu species	Position	%	Co species	Position	%
Cu^+^	932.47	32.04	Co^2+^	781.19	17.89
Cu^+^	952.28	37.21	Co^2+^	796.64	15.43
Total Cu^+^	69.25		Total Co^2+^	51.02	
Cu^2+^	934.32	21.37	Co^3+^	779.43	35.81
Cu^2+^	958.54	9.38	Co^3+^	794.64	30.87
Total Cu^2+^	30.75		Total Co^3+^	66.68	

Unlike the Cu 2*p*, the HR Co 2*p* XPS spectrum (Figure 4c) is sharp and much more resolved, which might be due to the amount of this element present on the surface when compared to the Cu ones (Table 1), also agreeing with EDS mappings. Deconvolution analysis of the HR Co 2*p* XPS spectrum also revealed a mixture of Co^2+^/Co^3+^ cations on the samples’ surface, which is commonly observed in Co‐containing materials.^[^ ^25^ ^,^ ^73^ ^–^ ^75^ ^]^ Surprisingly, weak satellite peaks were observed in the Co 2*p* XPS spectrum, confirming that a higher amount of Co^3+^ (66.68%) than reduced Co^2+^ species is present on the material surface.^[^ ^34^ ^]^ It is worth noting that the high concentration of Co^3+^ ions is ideal for OER, as it is well established in the literature that these ions serve as more active catalytic sites.^[^ ^76^ ^]^ The Cu^+^‐richer and Co^2+^‐containing surface is also associated with the presence of oxygen vacancies, which are formed during the synthesis of the material and influence the electrochemical and redox cycling, as noted.

To further confirm the presence of oxygen vacancies, the deconvolution of the HR O 1*s* XPS spectrum is also shown (Figure 4d, **Table** **3**), displaying peaks corresponding to lattice oxygen atoms (O^struct^) bonded to the metal, and adsorbed oxygen species of metal carbonates on oxygen vacancies (Vo). The presence of the photoemission signals of Cu, Co, and O elements in CuCo_2_O_4_ is consistent with other works reported in the literature,^[^ ^25^ ^,^ ^34^ ^,^ ^73^ ^]^ confirming that the target composition is successfully obtained in the present case. Apart from these, the presence of the C 1*s* signal (Figure 4e, **Table** **4**) suggests that residual carbon remains on the material's surface after calcination to obtain CuCo_2_O_4_, which is consistent with our previous works dealing with MOF‐derived oxide materials.^[^ ^75^ ^,^ ^77^ ^,^ ^78^ ^]^

**Table 3:** Deconvolution data of the HR O 1s XPS spectrum.

	CuCo_2_O_4_	
	Position	%
O^struct^/Metal oxide	529.34	34.86
Vo/Metal carbonate	530.91	65.14

**Table 4:** Deconvolution data of the HR C 1s XPS spectrum.

	CuCo_2_O_4_	
	Position	%
C—C	284.37	77.24
C—O—C	285.83	9.78
O—C=O	288.01	12.98

### 3.3 Electrochemical Performance

#### 3.3.1 Oxygen Evolution Reaction

**Figure 5:**
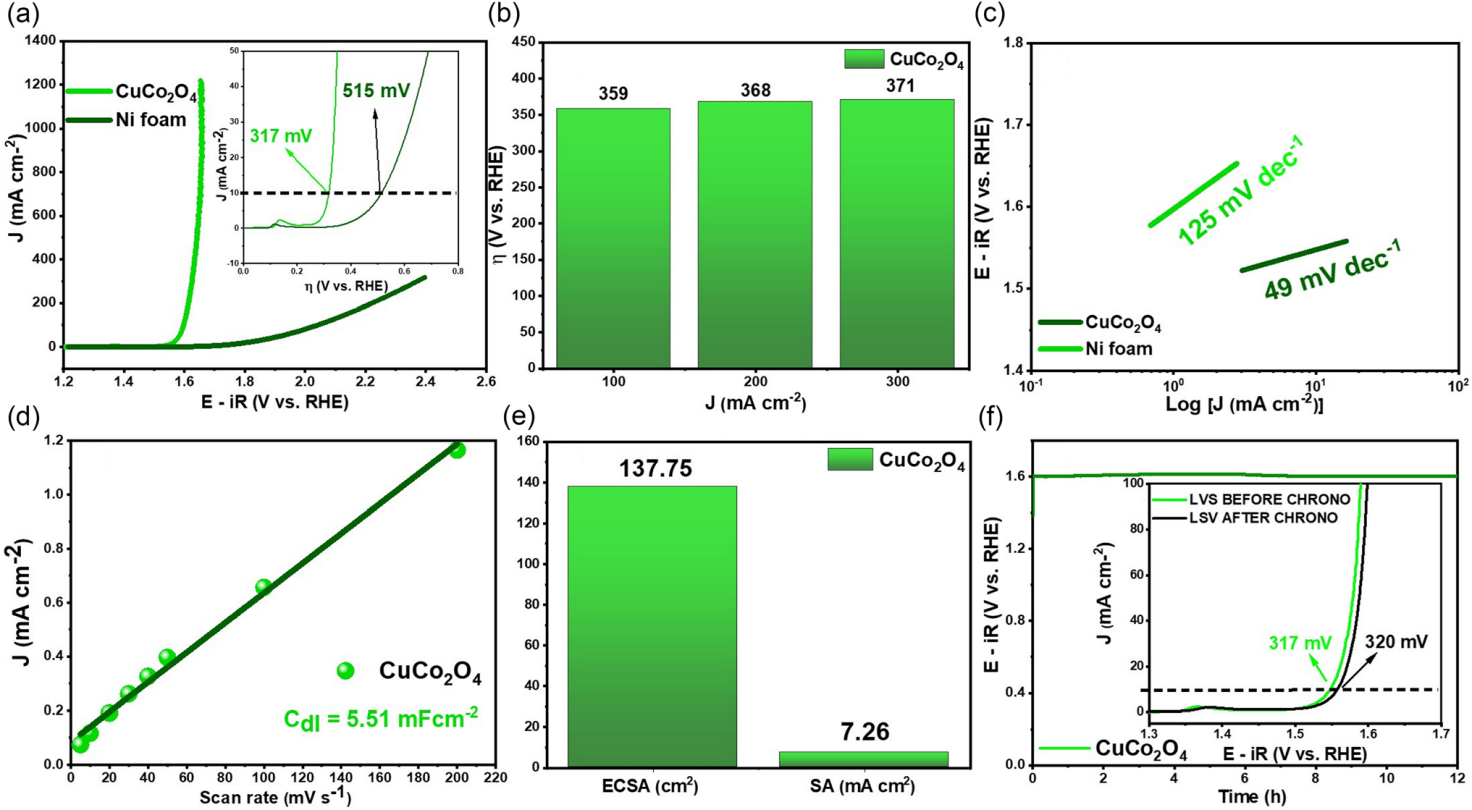
a) LSV curves, b) overpotential at different current densities, c) Tafel slopes, d) electrochemical double layer capacitance, e) electrochemical active area/specific activity, and f) chronopotentiometry test at *J* = 10 mA cm^*−*2^ for 12 h.

The electrocatalytic activity of the CuCo_2_O_4_ electrode for OER was evaluated using LSV, CV, CP, and EIS using 1.0 M KOH as electrolyte. The results were compared with those obtained from a Ni foam electrode, which is widely used as a support material due to its 3D structure, high conductivity and porosity, and low cost.^[^ ^79^ ^]^ **Figure** **5**a shows the polarization curves of CuCo_2_O_4_ and Ni foam, with overpotentials of 317 and 515 mV versus RHE at a current density of 10 mA cm^−2^, respectively. This result is superior to many cobaltite‐based electrodes reported in the literature (Table S1, Supporting Information). For comparison, Ashiq et al.^[^ ^80^ ^]^ reported an overpotential *η* = 364 mV for CuO, while Farid et al*.* ^[^ ^81^ ^]^ obtained 400 mV for Co_3_O_4_ synthesized by carbonization of ZIF‐67. Although these materials exhibit different morphologies, the literature indicates a significant improvement in the electrocatalytic performance of CuCo_2_O_4_. Pure CuO and Co_3_O_4_ (for comparison) obtained from the calcination of pure ZIF‐67 and pure CuIDA at 350 °C exhibited overpotentials of 359 mV (Figure S10a, Supporting Information) and 385 mV (Figure S10c, Supporting Information), respectively. Figure 5b shows the electrochemical performance of CuCo_2_O_4_ over the entire current density range. Even at a high current density of 100 mA cm^−2^, CuCo_2_O_4_ still showed superior performance, with *η*_100_ equal to 359 mV, compared to 508 mV for the pristine CuO and 529 mV for the bare Co_3_O_4_.

Reaction kinetics were evaluated via Tafel slope, calculated from the linear relationship *η* = *a* + *b* log*j*_0_, where *η* is the overpotential, *a* is the intercept related to the exchange current density (*j*_0_), and *b* is the Tafel slope.^[^ ^82^ ^]^ Tafel plots (Figure 5c) indicated Tafel slope of only 49 mV dec^−1^, far lower compared to the pristine CuO (82.5 mV dec^−1^, Figure S10b, Supporting Information) and pure Co_3_O_4_ (114.12 mV dec^−1^, Figure S8d, Supporting Information), obtained from the bare MOFs CuIDA and ZIF‐67, respectively. Considering the well‐known Krasil'shchikov kinetic model for OER (following equations),^[^ ^83^ ^]^ experimental Tafel slopes suggest a superior reaction kinetics for the CuCo_2_O_4_ electrocatalyst. In this way, the reaction $M^{*}O^{-}\to M^{*}O+e^{-}$ as the main rate‐determining step, since the experimental value closely matches the expected Tafel slope for this reaction step.

(3) $$\begin{array}{l} M^{*}+{\mathrm{HO}}^{-}\to M^{*}\mathrm{OH}+e^{-}b=120{\text{mV dec}}^{-1}\\ M^{*}\mathrm{OH}+{\mathrm{HO}}^{-}\to M^{*}O^{-}+H_{2}Ob=60{\text{mV dec}}^{-1}\\ M^{*}O^{-}\to M^{*}O+e^{-}b=45{\text{mV dec}}^{-1}\\ 2M^{*}O\to 2M^{*}+O_{2}b=19{\text{mV dec}}^{-1} \end{array}$$

The double layer capacitance (*C*_dl_) for the CuCo_2_O_4_ electrocatalyst was obtained via CV data in a non‐faradaic (Figure S11, Supporting Information), through the relationship between current density and scan rate ($j_{c}=v\times C_{\mathrm{dl}}$), shown in Figure 5d. Experimental *C*_dl_ was 5.51 mF, and electrochemically active surface area (ECSA) was calculated using Equation (4)^[^ ^84^ ^]^

(4) $$\text{ECSA}=\frac{C_{\mathrm{dl}}}{C_{s}}$$

where *C*_s_ is the specific capacitance, with value of 0.04 mF cm^−2^ for a transition metal‐based OER electrocatalyst in KOH solution.^[^ ^85^ ^]^ The calculated ECSA value for CuCo_2_O_4_ was 137.75 cm^2^, indicating a large active surface area and highly exposed active sites, which enhances the OER activity.^[^ ^86^ ^,^ ^87^ ^]^ Although the ECSA value obtained is considerably high, it only reflects the potential active sites available for the electrocatalytic process. Table S1, Supporting Information, presents a comparison of overpotential and ECSA values for similar materials reported in the literature. While there is no direct proportional relationship between these values, in general, higher ECSAs are observed in electrocatalysts exhibiting lower overpotentials. It is worth noting that the value obtained in this work is comparable to those reported for other cobaltites with similar overpotential, as shown in Table S1, Supporting Information. The CP test at *j* = 10 mA cm^−2^ (Figure 5f) indicates a high stability up to 12 h, with no significant decline in current density. No significant changes were observed in the overpotential after the CP experiments were observed (inset Figure 5f), showing the good electrochemical stability of CuCo_2_O_4_. Based on the above results, it can be confirmed that the CuCo_2_O_4_ electrode is a promising candidate for OER.

**Figure 6:**
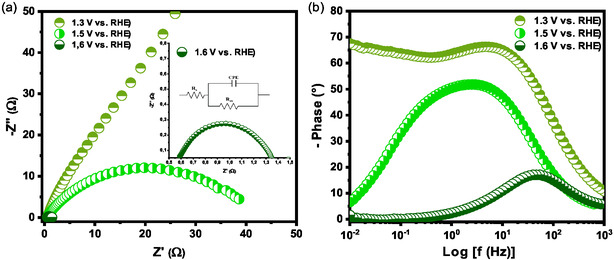
a) Nyquist and b) Bode plots of the CuCo_2_O_4_.

**Figure** **6** shows the Nyquist and Bode plots for CuCo_2_O_4_ at three potentials to investigate the performance of the electrocatalyst before, during, and after OER. The diameter of the semicircle in the Nyquist plot is indicative of the charge transfer resistance at the electrode surface. A larger semicircle indicates higher resistance to charge transfer at the interface between the electrocatalyst and the electrolyte.^[^ ^88^ ^]^ At 1.3 V versus RHE, a potential is below the onset of the oxygen evolution reaction, and an incomplete semicircle was observed. In contrast, the data at 1.5 and 1.6 V versus RHE were successfully fitted using a simplified Randles circuit (inset in Figure 6a), which includes two resistances—electrolyte resistance (*R*_s_) and charge transfer resistance (*R*_ct_)—and a constant phase element (CPE) representing the double layer capacitance (*C*_dl_) phenomenon. The CPE impedance is given by $Z_{\mathrm{CPE}}={\left[Q_{\mathrm{CPE}}{\left(i\omega \right)}^{n}\right]}^{-1}$, where *n* is an exponent that varies from 0 to 1 and is used to determine the true capacitance through the equation $C=R_{\mathrm{ct}}^{(1-n)/n}Q^{1/n}$, which corresponds to the depression of the semicircle.^[^ ^89^ ^]^ The values obtained are listed in **Table** **5**. The *R*_s_ values remained constant across the three potentials, while *R*_ct_ decreased significantly with increasing potential, thereby enhancing OER kinetics. Additionally, the value of *C*_dl_ decreased with increasing potential, which is probably related to bubble formation on the electrode surface. The Bode plot (Figure 6b) indicates that the process is facilitated, occurring more rapidly as the potential increases. This is consistent with the observed decrease in *R*_ct_.

**Table 5:** Data from the ESI fitting spectra.

Electrocatalyst	*R*_s_ [Ω]	*R*_ct_ [Ω]	C [mF]	*n*
1.3	0.51	192.4	10.6	0.79
1.5	0.58	38.2	48.4	0.72
1.6	0.56	0.81	7.8	0.76

#### 3.3.2 Supercapacitor Applications

**Figure 7:**
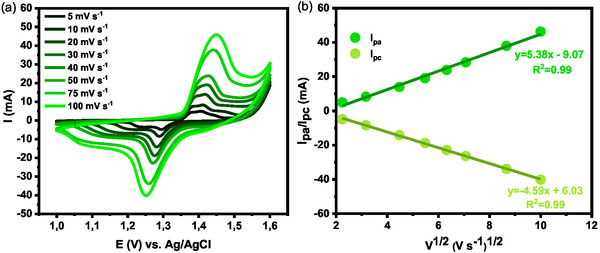
a) CV curves of the CuCo_2_O_4_ electrode measured at different scan rates in 1.0 M KOH. b) Plots of *i*_p_ versus v^1/2^ used to calculate the slopes for the anode and cathode sweeps.

Electrochemical analysis of the electrodes for SC applications was performed in a 1.0 M KOH solution within a fixed potential window of 0.0–0.6 V and at scan rates of 5–100 mV s^−1^ (**Figure** **7**a). The CV curves for the CuCo_2_O_4_ electrode displayed a pair of distinct redox peaks, which characterize battery‐like behavior.^[^ ^73^ ^]^ The well‐defined redox peaks indicate that the observed battery‐like behavior is associated with the redox transitions of cobalt and copper. As the scan rate increases, the redox peaks shift to more extreme values. This shift is due to the kinetic limitations of the electrochemical reactions; at higher scan rates, there is less time for the redox processes to occur, which requires higher potentials for oxidation and lower potentials for reduction. This results in the oxidation peaks moving toward more positive potential and the reduction peaks shifting toward more negative potential. In Figure 7b, a good linear relationship was observed between both the anodic and cathodic peak currents with the square root of the scan rate. This indicates that the electrode behavior corresponds to a diffusion‐controlled electrochemical process, demonstrating good stability of the alkaline electrolyte with the electrode material for energy storage applications.^[^ ^90^ ^,^ ^91^ ^]^

**Figure 8:**
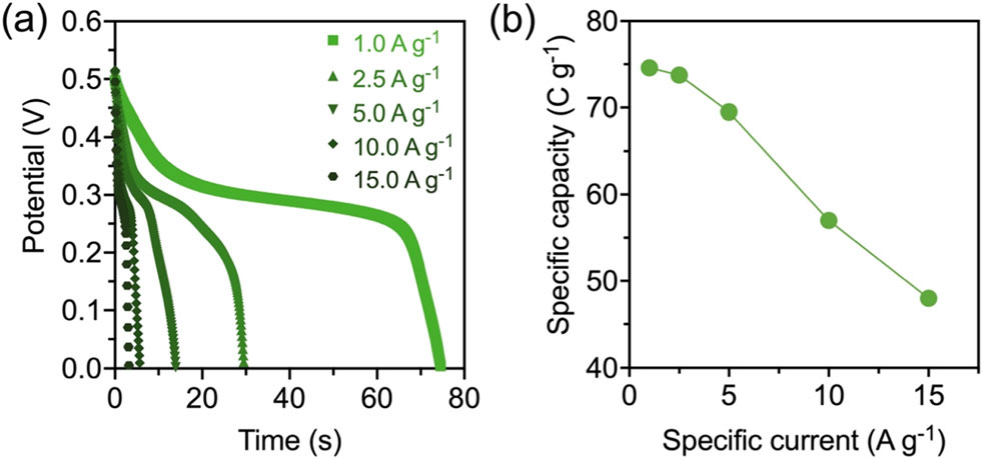
a) Discharge curves of the CuCo_2_O_4_ electrode measured at different specific currents in 1.0 M KOH, and b) specific capacity at specific currents ranging from 1 to 15 A g^*−*1^.

Galvanostatic charge–discharge (GCD) tests of CuCo_2_O_4_ were carried out within the potential range of 0–0.5 V, using specific currents ranging from 1 to 15 A g^−1^. The discharge plateau demonstrates the battery behavior of the synthesized CuCo_2_O_4_ electrode, which can be attributed to the capacity resulting from faradaic reactions. These findings are consistent with the observations made in the CV profiles (Figure 7). As shown in **Figure** **8**, at a specific current of 1 A g^−1^, the discharge time is ≈75 s, corresponding to a specific capacity (*C*_s_) of ≈75 C g^−1^, as calculated using Equation (3).^[^ ^92^ ^]^ As the current increases, the *C*_s_ tends to decrease due to limitations in the ion transport and increased internal resistance.^[^ ^93^ ^,^ ^94^ ^]^ Moreover, CuCo_2_O_4_ outperforms the reference samples, CuO and Co_3_O_4_, which exhibit specific capacities of ≈27 and ≈24 C g^−1^, respectively, at 1 A g^−1^ (Figure S12, Supporting Information). This suggests that the incorporation of copper into the spinel structure of cobalt oxide significantly enhances energy storage performance.

## 4 Conclusions

This study presents an innovative approach for synthesizing the bimetallic oxide CuCo_2_O_4_ through the thermal treatment of the ZIF‐67/CuIDA composite. The proposed synthetic route effectively produced CuCo_2_O_4_ with enhanced electrochemical properties, making it suitable for dual applications as OER catalyst and an electrode supercapacitor devices. The incorporation of copper into the spinel structure of cobalt oxide resulted in improved catalytic activity and energy storage performance compared to pure Co_3_O_4_ and CuO. Specifically, CuCo_2_O_4_ exhibited a lower overpotential for OER (317 mV at 10 mA cm^−2^) and a smaller Tafel slope (49 mV dec^−1^), indicating superior catalytic efficiency. Moreover, the material demonstrated excellent stability during 12 h of continuous operation. In terms of energy storage, the CuCo_2_O_4_ electrode showed a significantly higher specific capacity (≈75 C g^−1^ at 1 A g^−1^) than the reference samples, with capacities of ≈27 and ≈24 C g^−1^ for CuO and Co_3_O_4_, respectively. This indicates that the integration of copper in the spinel structure of cobalt oxide enhances its energy storage capabilities, making it a promising material for future energy applications.

## Conflict of Interest

The authors declare no conflict of interest.

## Author Contributions

**Johnnys da Silva Hortêncio:** conceptualization, methodology, validation, investigation, writing original draft, formal analysis. **Rafael A. Raimundo:** methodology, validation, investigation, formal analysis. **Allan J. M. Araújo:** methodology, validation, investigation, formal analysis. **André Luiz Menezes de Oliveira:** methodology, validation, investigation, formal analysis. **Daniel A. Macedo:** conceptualization, visualization, investigation, writing original draft, formal analysis, funding acquisition, project administration. **Sherlan Guimarães Lemos:** conceptualization, visualization, investigation, writing original draft, formal analysis, funding acquisition, project administration. **Fausthon Fred da Silva:** conceptualization, methodology, validation, resources, visualization, investigation, writing original draft, formal analysis, funding acquisition, project administration.

## Supporting information

Supplementary Material

## Data Availability

The data that support the findings of this study are available from the corresponding author upon reasonable request.
