# Supplementary material for: Bifunctional MOF‐on‐MOF‐Derived CuCo2O4 for Oxygen Evolution Reaction Electrocatalysis and Supercapacitor Electrodes
Source: ChemistryOpen. 2025 Jul 31;14(12):e202500180. doi: 10.1002/open.202500180 (PMC12680583; doi:10.1002/open.202500180)
Supplement: Supplementary file 1 — Supplementary Material [file OPEN-14-e202500180-s001.pdf]

Supplementary Information for:

Bifunctional MOF-on-MOF-derived $CuCo_2O_4$ for Oxygen Evolution Reaction Electrocatalysis and Supercapacitor Electrodes

Johnnys da Silva Hortêncio,[a] Rafael A. Raimundo,[b] Allan J. M. Araújo,[b,c] André Luiz Menezes de Oliveira,[d,e] Daniel A. Macedo,[f] Sherlan Guimarães Lemos,[a] Fausthon Fred da Silva,[a*]

[a] *Departamento de Química, Universidade Federal da Paraíba (UFPB), 58.051-900, João Pessoa – PB, Brazil.*
[b] *TEMA - Centre for Mechanical Technology and Automation, Department of Mechanical Engineering, University of Aveiro, 3810-193 Aveiro, Portugal.*
[c] *LASI - Intelligent Systems Associate Laboratory, 4800-058, Guimarães, Portugal.*
[d] Núcleo de Pesquisa e Extensão LACOM, Universidade Federal da Paraíba, 58051-900 Joãao Pessoa-PB, Brazil.
[e] Laboratório de Síntese Química de Materiais, Dept. de Engenharia de Materiais, Universidade Federal do Rio Grande do Norte, 59078-970 Natal-RN, Brazil
[e] *Programa de Pós-Graduação em Ciência e Engenharia de Materiais - PPCEM, Universidade Federal da Paraíba (UFPB), 58.051-900, João Pessoa, PB, Brazil.*
*Corresponding author: fausthon@quimica.ufpb.br

ORCIDs: 0000-0002-6095-512X (da Silva, F. F.); 0000-0003-0386-9329 (Allan J. M. Araújo)

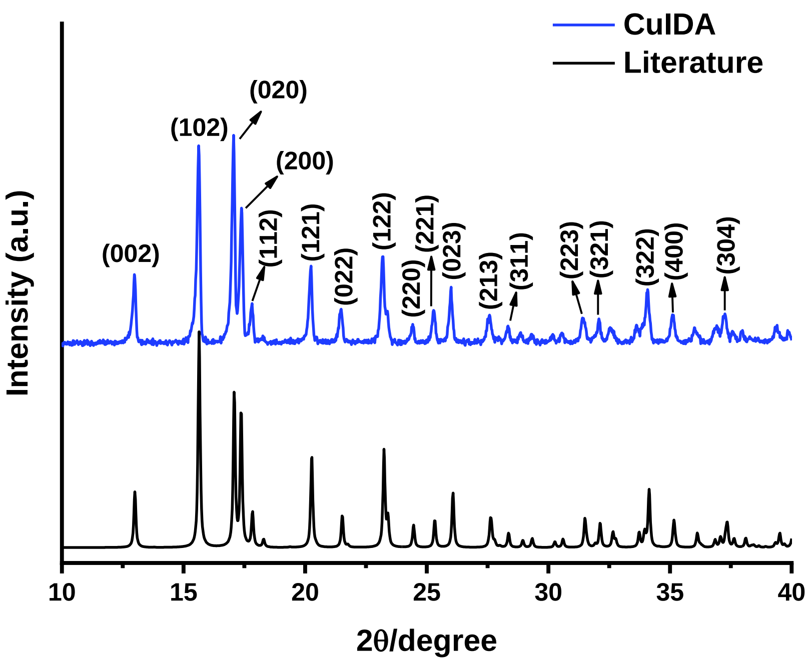

Fig. S1. Experimental powder diffraction pattern compared to literature (CIF 105855).

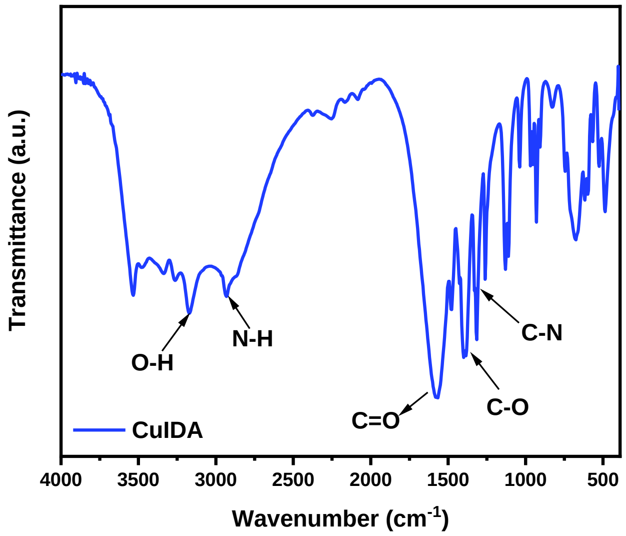

Fig. S2. CuIDA infrared spectrum.

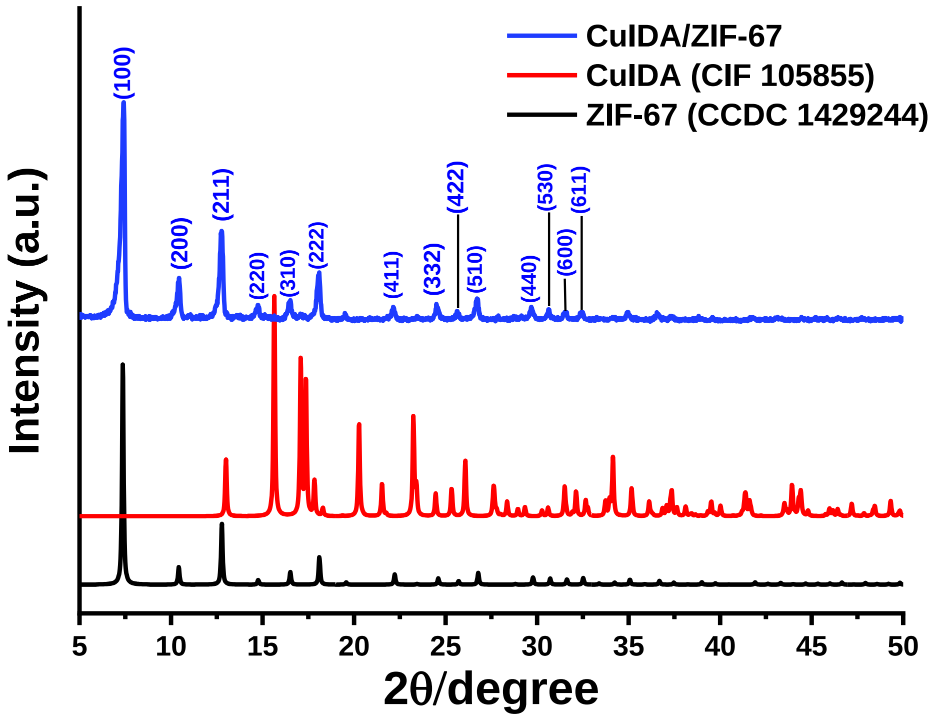

Fig S3. XRD of CuIDA/ZIF-67.

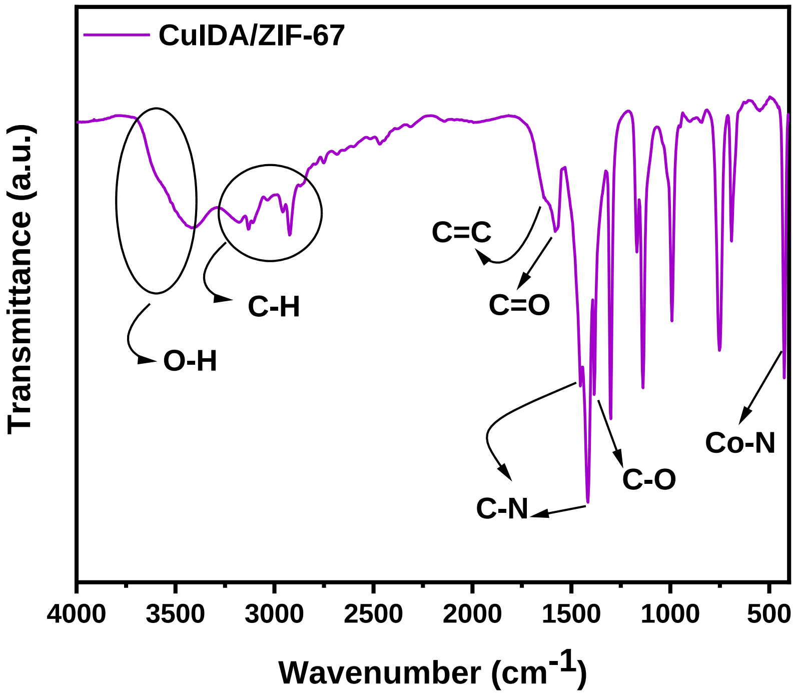

Fig. S4. Infrared spectrum of CuIDA/ZIF-67.

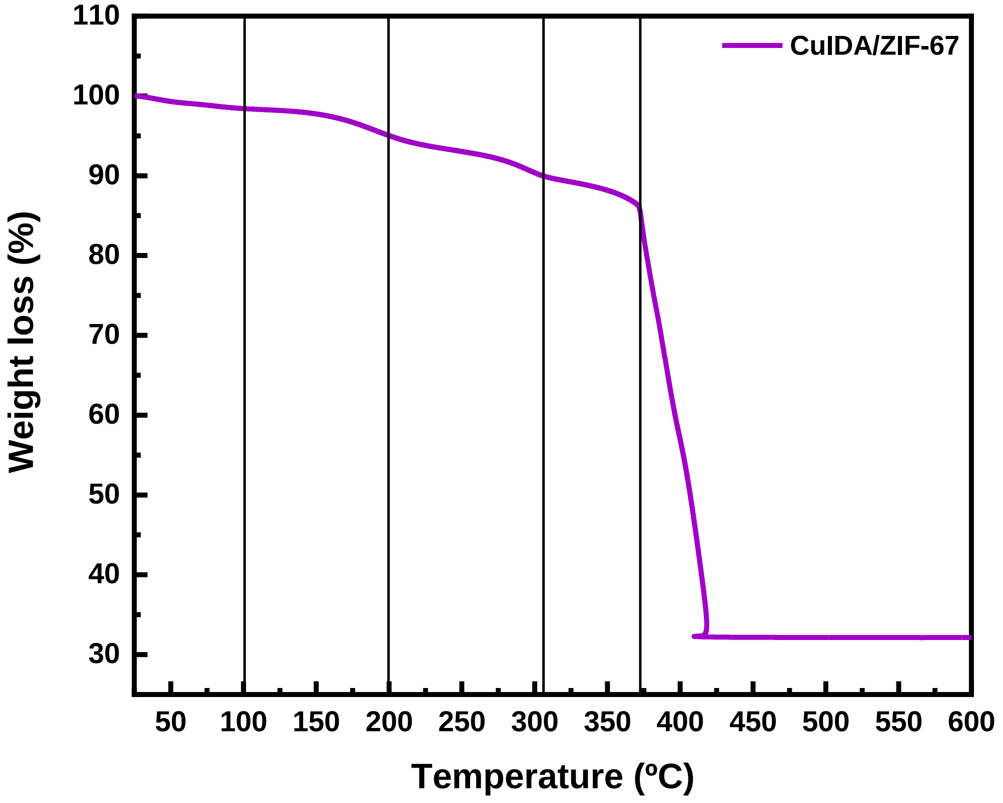

Fig. S5. Thermogravimetric curve for CuIDA/ZIF-67.

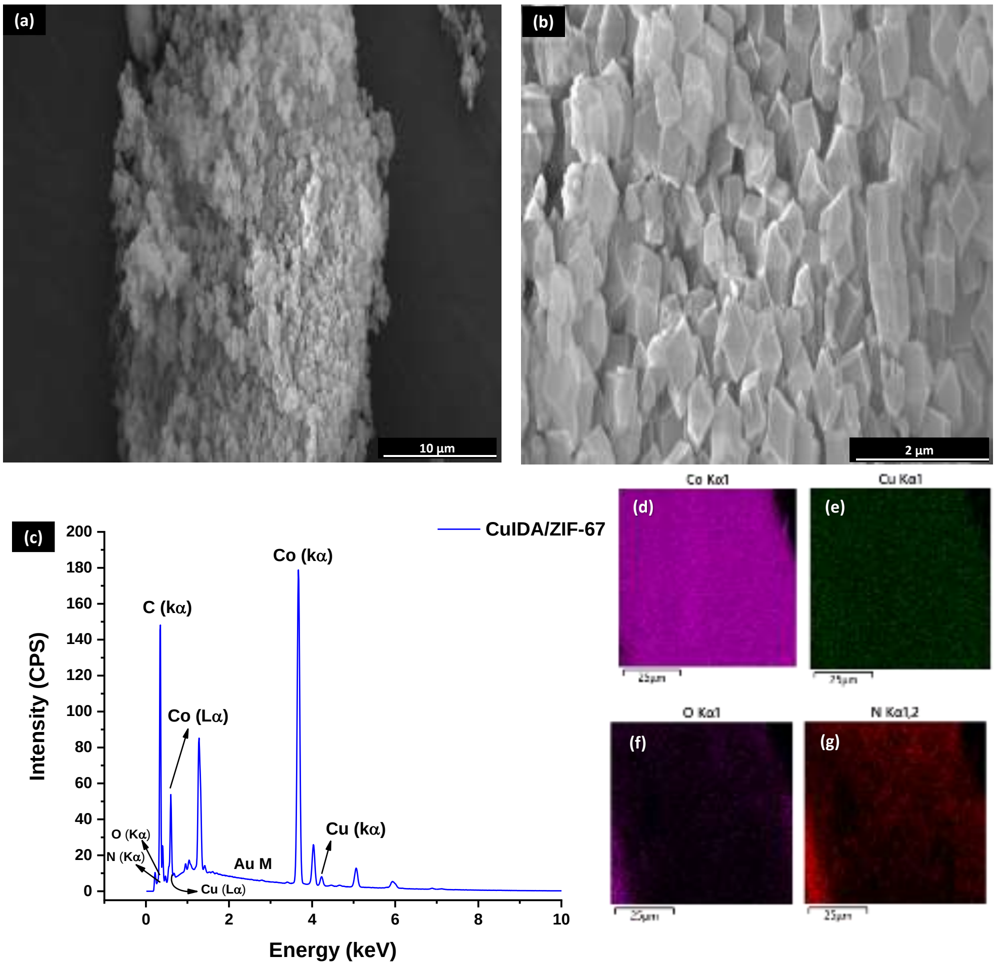

Fig. S6. SEM (a and b), (c) EDS spectrum, and (d-g) elemental mapping of CuIDA/ZIF-67.

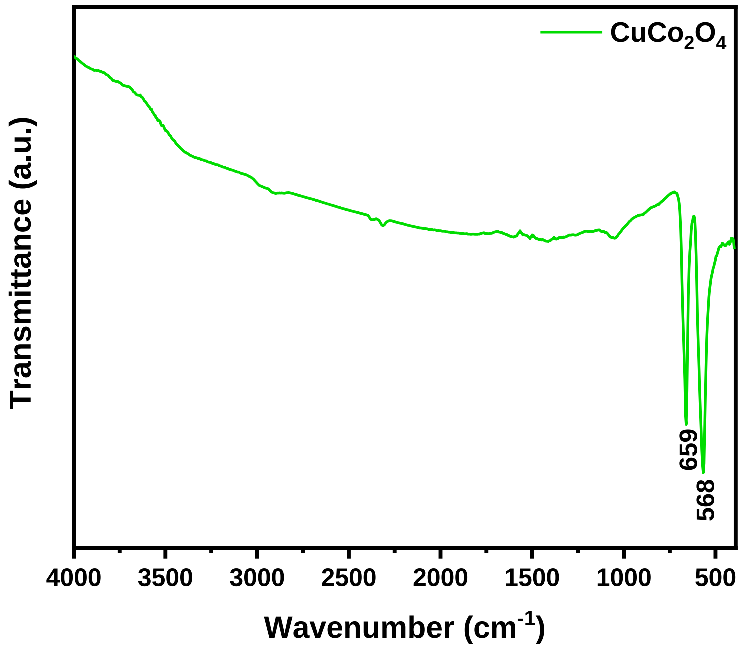

Figure S7. FTIR spectrum of $CuCo_2O_4$.

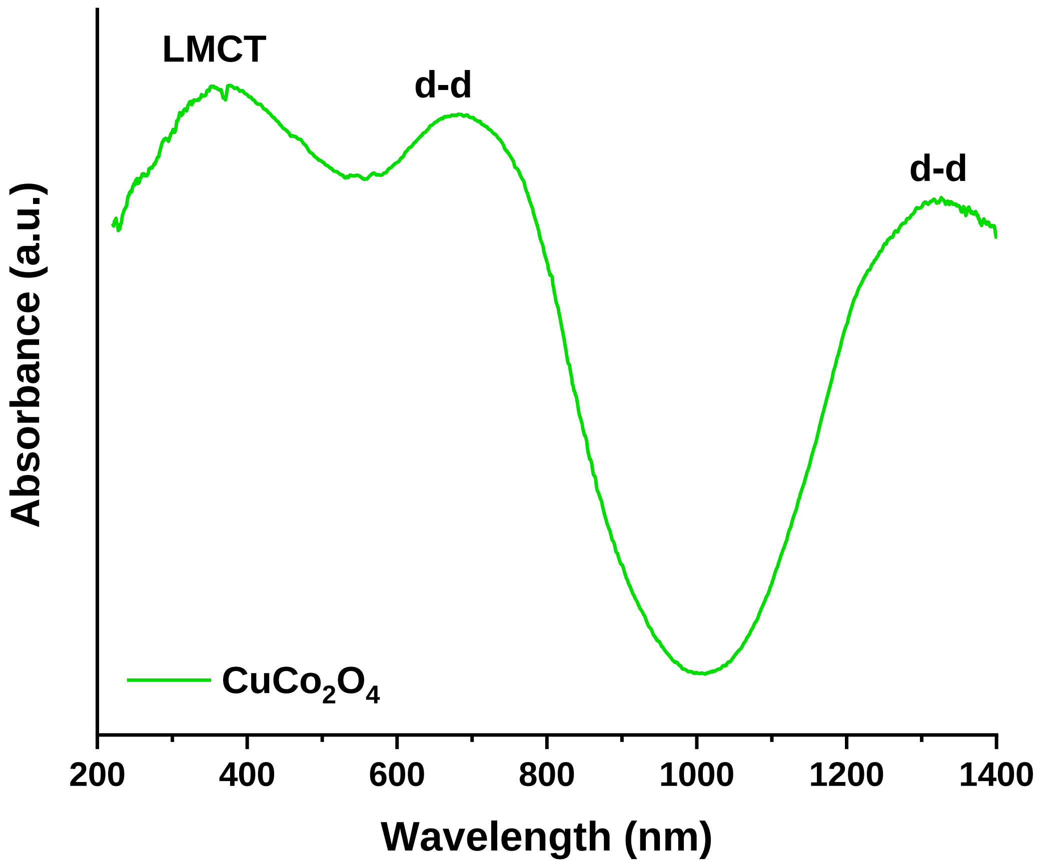

Fig. S8. Solid-state UV-VIS absorption spectrum of $CuCo_2O_4$.

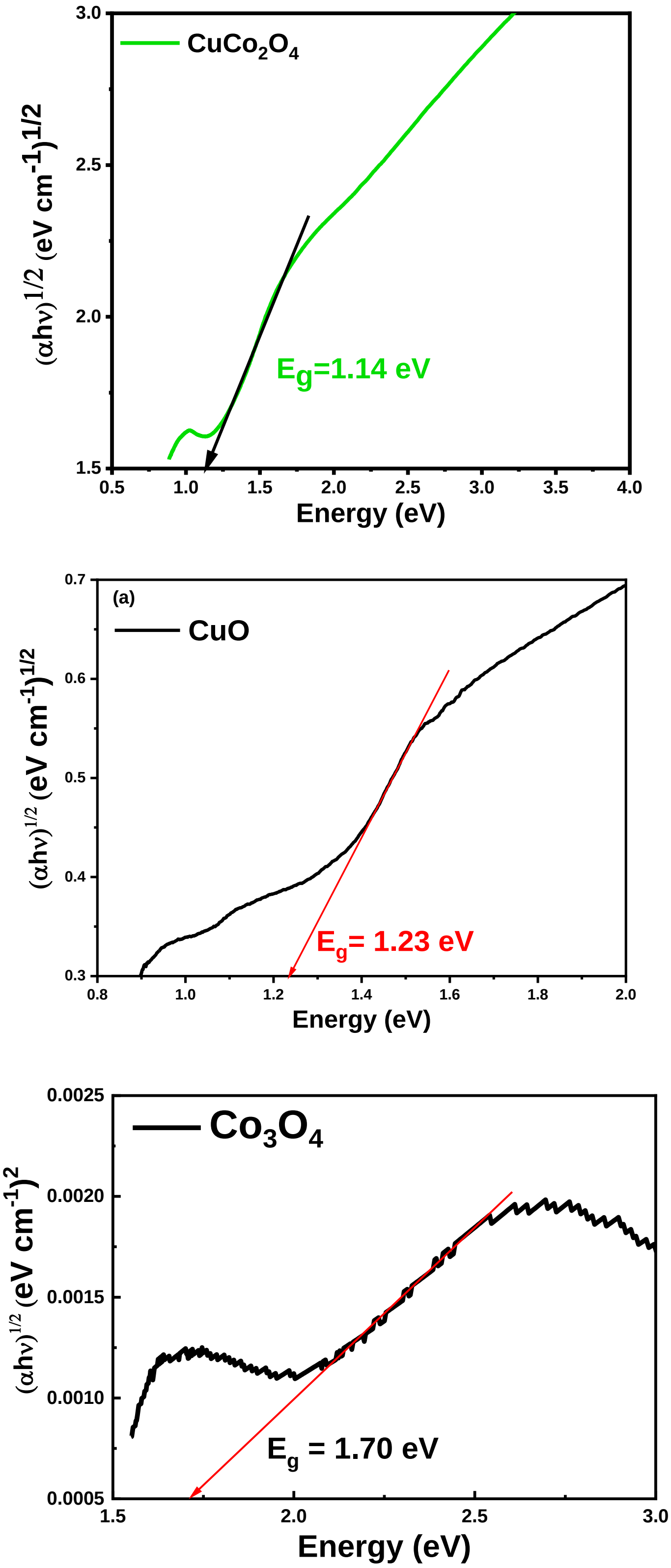

Fig. S9. Bandgap obtained by the Tauc plot method for $CuCo_2O_4$, and the pure CuO and Co_3O_4.

Table S1. Electrochemical performance of materials based on copper and cobalt for the OER in comparison to several oxides reported in the literature.

Electrocatalyst	η (mV) @10mAcm^{-2}	Tafel slope (mV dec^{-1})	ECSA (cm^{2})	Electrolyte	Ref.
$CuCo_2O_4$	317	49	137.75	1.0 M KOH	This work
$CuCo_2O_4$	460	101	--	1.0 M KOH	[1]
$CuCo_2O_4$/NrGO	360	64	4.9	1.0 M KOH	[2]
$CuCo_2O_4$	447	133.1	592.4	1.0 M KOH	[3]
$CuCo_2O_4$/CG	470	87	0.02	1.0 M KOH	[4]
$CuCo_2O_4$	470	67	1.8	0.1 M KOH	[2]
$CuCo_2O_4$/NF	364	159	570	1.0 M KOH	[5]
SnS/$CuCo_2O_4$/NF	348	122	707	1.0 M KOH	[5]
$CuCo_2O_4$ Polyhedron	330	90.3	--	1.0 M KOH	[6]
$CuCo_2O_4$/rGo nanoparticle	360	64	--	1.0 M KOH	[7]
$CuMn_{0.5}Co_2O_4$ nanoparticles	340	69.8	128	1.0 M KOH	[8]
Co_3O_4 nanoflawer	356	68	--	1.0 M KOH	[9]
Co_3O_4/CC	420	107	2.5	1.0 M KOH	[10]
$CuCo_2O_4$ nanochain	351	63.3	8.27	1.0 M KOH	[11]
$CuCo_2O_4$ polyhedron	420	90.3	71.5	1.0 M KOH	[12]
$NiCo_2O_4$ crystals	360	78.1	112.5	1.0 M KOH	[13]
$CuCo_2O_4$	346	75.9	0.25	1.0 M KOH	[14]
$CuCo_2O_4$	423	136	22	1.0 M KOH	[15]
$CuCe_{0.55}Co_{1.45}O_x$	352	79.5	0.1	1.0 M KOH	[14]
CeO_2/Co_3O_4	366	92.6	79.8	1.0 M KOH	[16]
Mn/ZIF-67C(E)	338	80.95	257.5	1.0 M KOH	[17]
Mn/ZIF-67C(M)	356	80.99	237.5	1.0 M KOH	[17]
$MnCo_2O_4$	400	190	--	1.0 M KOH	[18]
$MnCo_2O_4$	358	N.R	--	1.0 M KOH	[19]
$Co_{2.4}Mn_{0.6}O_4$	365	50.6	43.24	1.0 M KOH	[20]

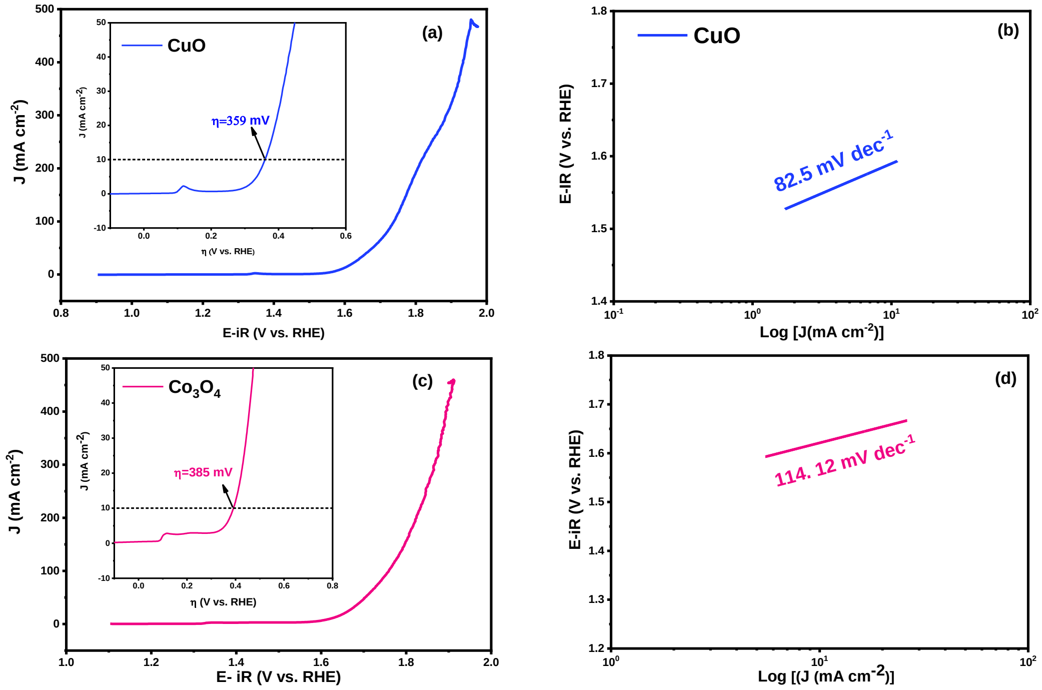

Fig. S10. (a) LSV curve for the CuO obtained from CuIDA; (b) Tafel slope for the CuO obtaneid from CuIDA; (c) LSV curve for the Co_3O_4 obtained from ZIF-67 and (d) Tafel slope for the Co_3O_4 obtained from ZIF-67.

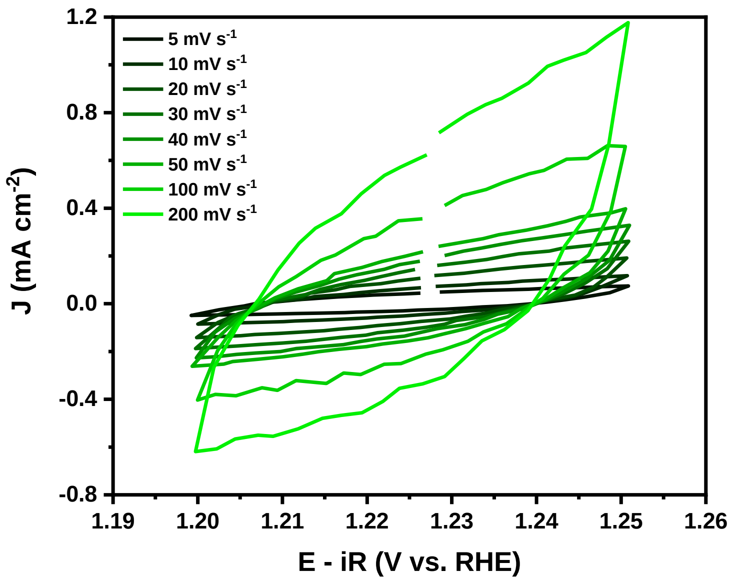

Fig. S11. Cyclic voltammetry curves for $CuCo_2O_4$.

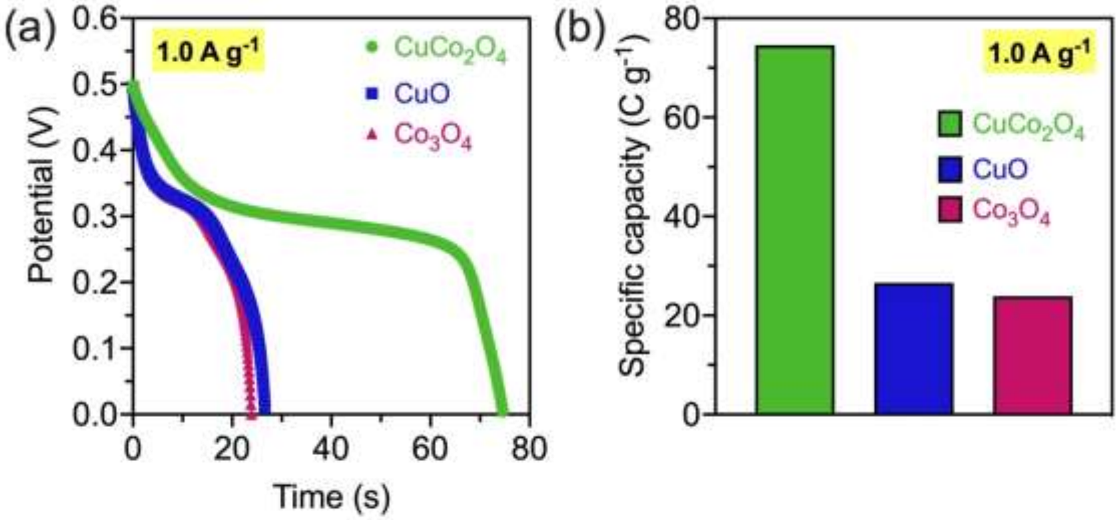

Fig. S12. (a) Discharge curves for $CuCo_2O_4$, CuO and Co_3O_4; b) Corresponding specific capacities at 1 A g^{-1}.

REFERENCES

[1] Y. Zhao, X. Zhou, Y. Ding, J. Huang, M. Zheng, and W. Ye, "A study of photocatalytic, chemical, and electrocatalytic water oxidation on ACo2O4 (A = Ni, Cu, Zn) samples through doping different metal ions," *J Catal*, vol. 338, pp. 30–37, Jun. 2016, doi: 10.1016/j.jcat.2016.02.003.

[2] S. K. Bikkarolla and P. Papakonstantinou, "CuCo 2 O 4 nanoparticles on nitrogenated graphene as highly efficient oxygen evolution catalyst," *J Power Sources*, vol. 281, pp. 243–251, May 2015, doi: 10.1016/j.jpowsour.2015.01.192.

[3] X. Ding, J. Liu, R. Cang, X. Chang, and M. Zhang, "Electrospun Hollow Carbon Nanofibers Decorated with CuCo2O4 Nanowires for Oxygen Evolution Reaction," *Catalysts*, vol. 12, no. 8, p. 851, Aug. 2022, doi: 10.3390/catal12080851.

[4] S. Ghorbanzadeh, S. A. Hosseini, and M. Alishahi, "CuCo2O4/Ti3C2Tx MXene hybrid electrocatalysts for oxygen evolution reaction of water splitting," *J Alloys Compd*, vol. 920, p. 165811, Nov. 2022, doi: 10.1016/j.jallcom.2022.165811.

[5] G. John, T. Susikumar, V. G. Sree, M. Navaneethan, and P. Justin Jesuraj, “Anchoring SnS nanoflakes on CuCo2O4 acicular sprouts for overall water splitting,” *Int J Hydrogen Energy*, vol. 51, pp. 1016–1027, Jan. 2024, doi: 10.1016/j.ijhydene.2023.07.139.

[6] S. K. Kaverlavani, S. E. Moosavifard, and A. Bakouei, “Designing graphene-wrapped nanoporous $CuCo_2O_4$ hollow spheres electrodes for high-performance asymmetric supercapacitors,” *J Mater Chem A Mater*, vol. 5, no. 27, pp. 14301–14309, 2017, doi: 10.1039/C7TA03943C.

[7] X. Zou, J. Su, R. Silva, A. Goswami, B. R. Sathe, and T. Asefa, “Efficient oxygen evolution reaction catalyzed by low-density Ni-doped Co3O4 nanomaterials derived from metal-embedded graphitic C3N4,” *Chemical Communications*, vol. 49, no. 68, p. 7522, 2013, doi: 10.1039/c3cc42891e.

[8] X. He *et al.*, “Tuning Electronic Structure of $CuCo_2O_4$ Spinel via Mn-Doping for Enhancing Oxygen Evolution Reaction,” *ChemElectroChem*, vol. 10, no. 2, Jan. 2023, doi: 10.1002/celc.202200933.

[9] C. K. Ranaweera *et al.*, “Flower-shaped cobalt oxide nano-structures as an efficient, flexible and stable electrocatalyst for the oxygen evolution reaction,” *Mater Chem Front*, vol. 1, no. 8, pp. 1580–1584, 2017, doi: 10.1039/C7QM00108H.

[10] G. Yang *et al.*, “Plasma enhanced atomic-layer-deposited nickel oxide on Co3O4 arrays as highly active electrocatalyst for oxygen evolution reaction,” *J Power Sources*, vol. 481, p. 228925, Jan. 2021, doi: 10.1016/j.jpowsour.2020.228925.

[11] A. Karmakar and S. K. Srivastava, “Interconnected Copper Cobaltite Nanochains as Efficient Electrocatalysts for Water Oxidation in Alkaline Medium,” *ACS Appl Mater Interfaces*, vol. 9, no. 27, pp. 22378–22387, Jul. 2017, doi: 10.1021/acsami.7b03029.

[12] G. Wei, J. He, W. Zhang, X. Zhao, S. Qiu, and C. An, “Rational Design of Co(II) Dominant and Oxygen Vacancy Defective $CuCo_2O_4$@CQDs Hollow Spheres for Enhanced Overall Water Splitting and Supercapacitor Performance,” *Inorg Chem*, vol. 57, no. 12, pp. 7380–7389, Jun. 2018, doi: 10.1021/acs.inorgchem.8b01020.

[13] L. Fang *et al.*, “Crystal-plane engineering of NiCo2O4 electrocatalysts towards efficient overall water splitting,” *J Catal*, vol. 357, pp. 238–246, Jan. 2018, doi: 10.1016/j.jcat.2017.11.017.

[14] W. Shi, Y. Zhang, L. Bo, X. Guan, Y. Wang, and J. Tong, “Ce-Substituted Spinel $CuCo_2O_4$ Quantum Dots with High Oxygen Vacancies and Greatly Improved Electrocatalytic Activity for Oxygen Evolution Reaction,” *Inorg Chem*, vol. 60, no. 24, pp. 19136–19144, Dec. 2021, doi: 10.1021/acs.inorgchem.1c02931.

[15] Z. Guo, Y. Pang, H. Xie, G. He, I. P. Parkin, and G. Chai, “Phosphorus-Doped $CuCo_2O_4$ Oxide with Partial Amorphous Phase as a Robust Electrocatalyst for the Oxygen Evolution Reaction,” *ChemElectroChem*, vol. 8, no. 1, pp. 135–141, Jan. 2021, doi: 10.1002/celc.202001312.

[16] A. L. de Souto Neto, A. de A. Lourenço, R. B. Silva, R. A. Raimundo, D. A. Macedo, and F. F. da Silva, “Metal-organic frameworks derived CeO2/Co3O4 nanocomposite as a new electrocatalyst for oxygen evolution reaction,” *Polyhedron*, vol. 238, p. 116390, Jul. 2023, doi: 10.1016/j.poly.2023.116390.

[17] A. A. Lourenço *et al.*, “Metal-organic frameworks as template for synthesis of Mn3+/Mn4+ mixed valence manganese cobaltites electrocatalysts for oxygen evolution reaction,” *J Colloid Interface Sci*, vol. 582, pp. 124–136, Jan. 2021, doi: 10.1016/j.jcis.2020.08.041.

[18] S. Natarajan, S. Anantharaj, R. J. Tayade, H. C. Bajaj, and S. Kundu, “Recovered spinel $MnCo_2O_4$ from spent lithium-ion batteries for enhanced electrocatalytic oxygen evolution in alkaline medium,” *Dalton Trans.*, vol. 46, no. 41, pp. 14382–14392, 2017, doi: 10.1039/C7DT02613G.

[19] D. M. Alqahtani *et al.*, “Effect of metal ion substitution on electrochemical properties of cobalt oxide,” *J Alloys Compd*, vol. 771, pp. 951–959, Jan. 2019, doi: 10.1016/j.jallcom.2018.09.014.

[20] K. R. Park *et al.*, “Synthesis of rod-type Co2.4Mn0.6O4 via oxalate precipitation for water splitting catalysts,” *Appl Surf Sci*, vol. 510, p. 145390, Apr. 2020, doi: 10.1016/j.apsusc.2020.145390.
